# *Rickettsia* spp. in rodent-attached ticks in Estonia and first evidence of spotted fever group *Rickettsia* species *Candidatus* Rickettsia uralica in Europe

**DOI:** 10.1186/s13071-020-04564-7

**Published:** 2021-01-20

**Authors:** Maria Vikentjeva, Julia Geller, Jaanus Remm, Irina Golovljova

**Affiliations:** 1 Department of Virology and Immunology, National Institute for Health Development, Tallinn, Estonia; 2 Department of Gene Technology, Tallinn University of Technology, Tallinn, Estonia; 3 Department of Zoology, University of Tartu, Tartu, Estonia; 4 Tallinn Children’s Hospital, Tallinn, Estonia

**Keywords:** Ticks, *Rickettsia* spp., *Candidatus* Rickettsia uralica, Voles, Mice

## Abstract

**Background:**

*Rickettsia* spp. are human pathogens that cause a number of diseases and are transmitted by arthropods, such as ixodid ticks. Estonia is one of few regions where the distribution area of two medically important tick species, *Ixodes persulcatus* and *I. ricinus*, overlaps. The nidicolous rodent-associated *Ixodes* *trianguliceps* has also recently been shown to be present in Estonia. Although no data are available on human disease(s) caused by tick-borne *Rickettsia* spp. in Estonia, the presence of three *Rickettsia* species in non-nidicolous ticks has been previously reported. The aim of this study was to detect, identify and partially characterize *Rickettsia* species in nidicolous and non-nidicolous ticks attached to rodents in Estonia.

**Results:**

Larvae and nymphs of *I.* *ricinus* (*n* = 1004), *I*. *persulcatus* (*n* = 75) and *I.* *trianguliceps* (*n* = 117), all removed from rodents and shrews caught in different parts of Estonia, were studied for the presence of *Rickettsia* spp. by nested PCR. Ticks were collected from 314 small animals of five species [*Myodes glareolus* (bank voles), *Apodemus flavicollis* (yellow necked mice), *A.* *agrarius* (striped field mice), *Microtus subterranius* (pine voles) and *Sorex araneus* (common shrews)]. Rickettsial DNA was detected in 8.7% (103/1186) of the studied ticks. In addition to identifying *R.* *helvetica*, which had been previously found in questing ticks, we report here the first time that the recently described *I.* *trianguliceps-*associated *Candidatus* Rickettsia uralica has been identified west of the Ural Mountains.
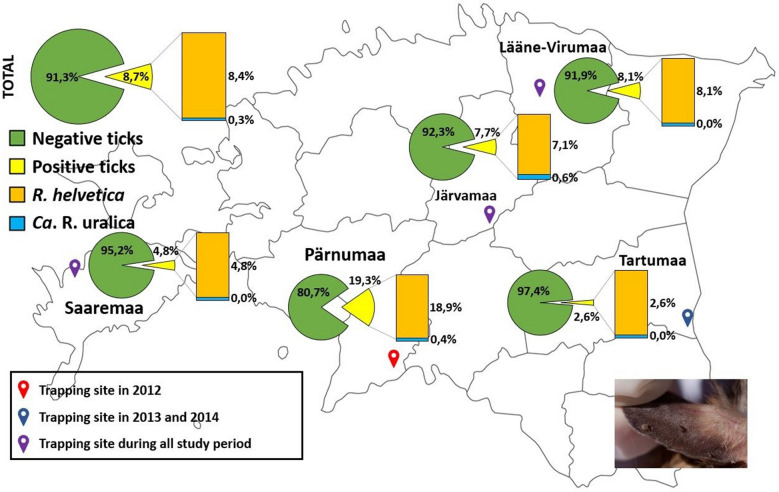

## Background

*Rickettsia* is a genus of small, obligate intracellular Gram-negative bacteria. Based on genomic analyses they are classified into four groups: the spotted fever group (SFG), the typhus group, the ancestral group and the transitional group [1]. Some SFG rickettsiae are transmitted by ticks of the family Ixodidae [2], and transmission may occur transovarially as well as transstadially [3, 4]. Several agents of tick-borne rickettsioses are known to circulate in Europe, including *Rickettsia conorii*, *R.* *massiliae*, *R.* *slovaca*, *R.* *raoultii*, *R.* *monacensis* and *R.* *helvetica* [2, 5], of which the last-mentioned is a frequently detected species in numerous Ixodidae ticks, including *Ixodes ricinus*, *I.* *persulcatus*, *I.* *trianguliceps* and *Dermacentor reticulatus* [2, 6, 7]. Although *R. helvetica* is not believed to be highly pathogenic to humans, several reports from Sweden [8, 9], the Netherlands [10], France and Italy [11] describe rash, mild fever, febrile illness, meningitis and other clinical symptoms associated with this agent in patients. In Estonia, Katargina et al. [12] reported the wide distribution of *R. helvetica*, as well as the presence of *R.* *monacensis* and *Candidatus* Rickettsia (*Ca.* R.) tarasevichiae, in questing ticks, but no human cases due to *R. helvetica* infection nor to the other two species had been reported at that time (2015).

Research on the circulation of *Rickettsia* spp. is still ongoing, btoh in vectors, which are mainly fleas and ticks, and in the latter’s main hosts (small mammals, wild and domestic animals). This is fairly wide research area, and new species are constantly being discovered, such as *I. trianguliceps*-associated *Ca.* R. uralica found in the Ural Mountains in Russia [7]. Also, the wide distribution of some types of vectors increases the probability of the prevalence of vector-associated pathogens, such as *Ca.* R. tarasevichiae, that have been found in China and Europe [12–14].

Current methods for disease surveillance include, among others, studying sentinel populations for the presence of pathogens in nature. Dogs [15] and cats [16] can be used as sentinels for rickettsiae. However, vectors can also serve as epidemiological sentinels [17].

Recent studies show that *Ixodes* spp. ticks can serve not only as vectors but also as the reservoir host of *R. helvetica*. At the present time there is no clear understanding of whether an mammal species is the host of *R. helvetica*, but rickettsial DNA has been found in the blood of wild animals, such as rodents, roe deer and wild boar [18], and domestic animals, such as dogs and cats [19]. Moreover, Burri et al. [20] reported negative results on *R. helvetica* xenodiagnostic as well as a low percentage of *Rickettsia* spp. from the positive host described by Tommassone et al. [21]. It can only be assumed that mammals can be potential hosts and that they may affect the natural transmission and distribution of rickettsiae.

The aim of the present study was to investigate the presence of *Rickettsia* spp. in ticks collected from small mammals.

## Methods

### Sample collection, species identification and DNA extraction

**Fig. 1:**
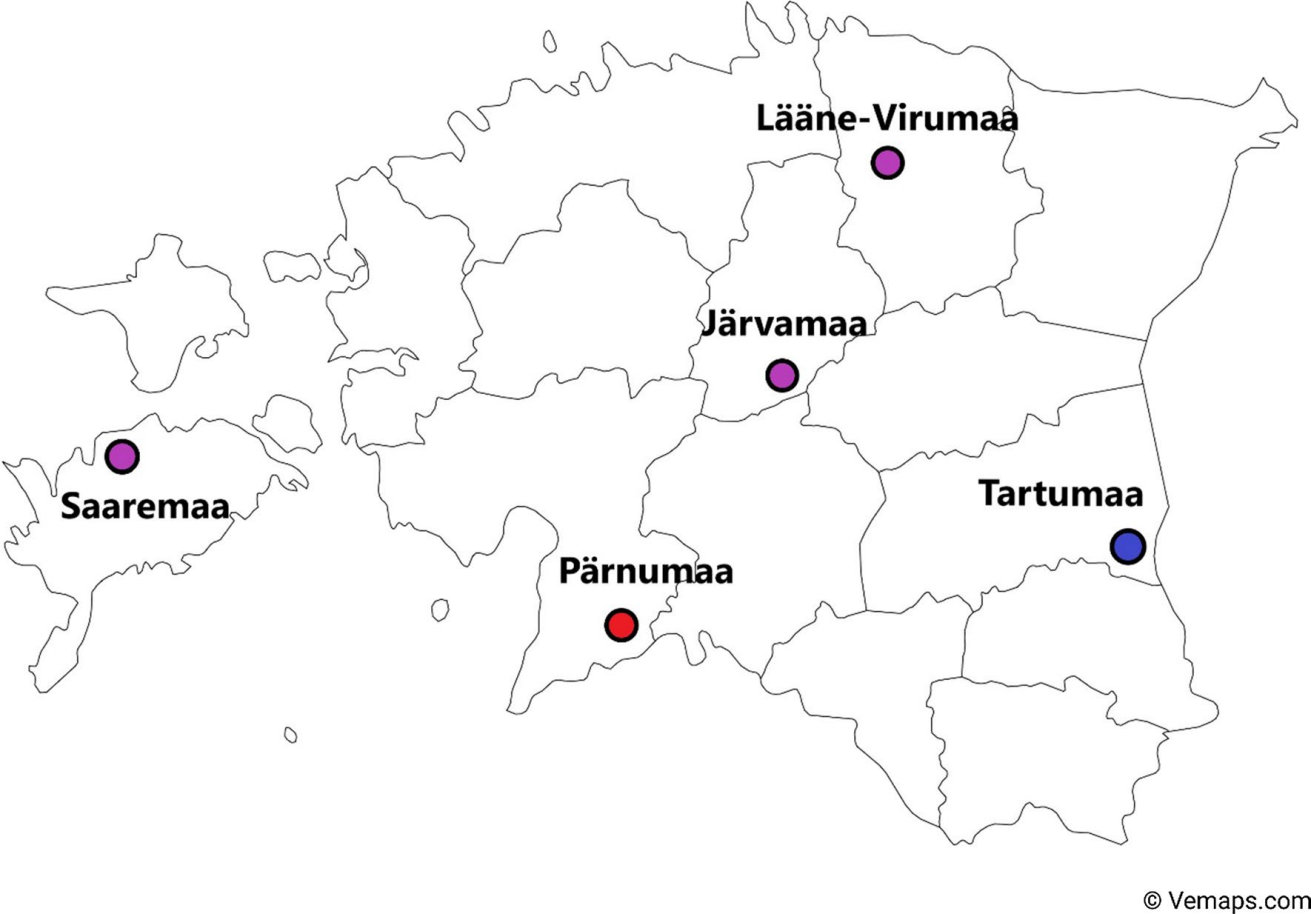
Trapping sites of small mammals during 2012–2014 in Estonia. Color of trapping sites indicated the study years: blue, trapping sites in 2013 and 2014; red, trapping sites in 2012; purple, trapping site in 2012–2014. The coordinates of the trapping sites are: Järvamaa (58.7365°N; 25.6682°E), Lääne-Virumaa (59.2260°N; 26.1335°E), Tartumaa (58.2493°N; 27.3023°E), Pärnumaa (58.0687°N; 24.8433°E) and Saaremaa (58.5075°N; 22.4107°E)

The study was performed retrospectively on 1186 ticks that had been removed from small mammals. The samples were collected at five sampling sites in Estonia, located in four mainland counties, namely Järvamaa, Lääne-Virumaa, Tartumaa (collecting in 2013 and 2014) and Pärnumaa (collecting was performed only in 2012), and in one island county, Saaremaa (Fig. 1). Live-trappings of mice, voles and shrews were carried out once a month during April–November 2012–2014 in natural habitats using Sherman LFA perforated live-traps (Ethical Committee Permission No. 124 by Estonian Ministry of Agriculture). Ten permanent stations (5 traps each station within a 2-m radius) were placed 100 m apart along a linear transect that randomly intersected different habitats (forest and semi-open). Trapping was performed during the nighttime, with the traps were set at 8 p.m. checked for animal the following morning at around 8 a.m. Bread was used as main bait method and vegetables served as water replenishment. The trapped animals were first identified to the species level and then killed by cervical dislocation by a specially trained person in accordance with Federation of European Laboratory Animal Science Association (FELASA) guidelines. Each animal was individually examined for the presence of ectoparasites, which were then removed, fixed in ethanol and stored at − 20 °C in separate tubes until further use. Any endangered species caught were immediately released in the habitat. For safety purposes, protective gloves and face masks were worn at all times while handling wild animals.

DNA was extracted from ticks using an ammonium hydroxide solution according to Moran-Cadenas et al. [22]. Tick species were identified by an internal transcribed spacer 2 gene (*ITS2*)-based multiplex PCR assay, as previously described by Värv et al. [23]. Only ticks identified at the species level based on *ITS2* multiplex PCR results were included in this study, and ticks whose species identity remained undetermined were not used in the study.

### *Rickettsia* spp. screening and genospecies detection

All ticks identified at the species level were screened individually by a nested PCR targeting a 667-bp fragment of the *Rickettsia* spp. citrate synthase A gene (*gltA*) using primers glt1–4, as described by Igolkina et al. [7], with subsequent sequencing of all positive samples. For samples identified as *Ca.* R. uralica and randomly selected samples identified as *R.* *helvetica* by initial screening, additional PCR amplification of ~ 770-bp fragment of the outer membrane protein B gene (*ompB*) was performed with primers 120-2788F and 120-3599R under conditions described previously [24]. Additionally, a subset of the latter samples was amplified by nested PCR of an 834-bp fragment of the cell-surface antigen 4 gene (*sca4*) with primers sc4-1 and Rj2837r for the primary reaction, and sc4-3 and sc4-4 for the nested reaction, as described by Igolkina et al. [7]. PCR products of all positive samples were sent for direct sequencing to the core laboratory of the Estonian Biocentre (Tartu, Estonia), followed by nucleotide sequence alignment using BioEdit v7.2.5 (Ibis Biosciences, Carlsbad, CA, USA) and genospecies identification with BLASTN® tools (http://www.ncbi.nlm.nih.gov/BLAST.cgi).

## Results

### *Rickettsia* screening and *Rickettsia* species

In this study 1186 ticks were collected from 314 small animals belonging to five mammalian species: *Myodes glareolus*, *Apodemus flavicollis*, *A. agrarius*, *Microtus subterranius* and *Sorex araneus* (Table 1; Additional file 1: Table S1). A total of 993 *I.* *ricinus* (924 larvae and 69 nymphs; from all 5 mammalian species), 117 *I.* *trianguliceps* (93 larvae and 24 nymphs, from *My. glareolus* and *A. flavicollis*) and 76 *I.* *persulcatus* (64 larvae and 12 nymphs, from *My. glareolus*, *A. flavicollis* and *M. subterranius*) were studied for presence of *Rickettsia* spp. (Table 2).

**Table 1:** Presence of *Rickettsia* spp. in different tick species attached to mice, voles and shrews collected from five Estonian sites (four mainland and one island counties)

County in Estonia where the small mammals were collected	Tick species attached to small mammals^a^											
	*Myodes glareolus* (bank vole)				*Apodemus flavicollis* (yellow necked mouse)				*Sorex araneus* (common shrew)			
	*IR*	*IT*	*IP*	Total (%)	*IR*	*IT*	*IP*	Total	*IR*	*IT*	*IP*	Total
Järvamaa	21/217 [﻿*Rh*]	2/16 [*Ca*Ru^b^]	0/1	23/234 (9.8%)	2/75 [*Rh*]	0/6	–	2/81 (2.5%)	0/10	–	–	0/10
Lääne-Virumaa	7/80 [﻿*Rh*]	1/22 [*Rh*^c^]	–	8/102 (7.8%)	9/83 [*Rh*]	0/23	0/1	9/107 (8.4%)	–	–	–	–
Tartumaa	1/23 [﻿*Rh*]	0/18	0/59	1/100 (1%)	3/32 [*Rh*]	0/5	0/13	3/50 (6%)	–	–	–	–
Pärnumaa	28/108 [﻿*Rh*]	1/13 [*Ca*Ru^b^]	0/1	29/122 (23.8%)	8/72 [*Rh*]	0/12	–	8/84 (9.5%)	7/21 [*Rh*^d^]	–	–	7/21 (33.3%)
Saaremaa	8/111 [﻿*Rh*]	0/1	–	8/112 (7.1%)	5/140 [*Rh*]	0/1	–	5/141 (3.5%)	–	–	–	–
Total	65/539 (12.1%)	4/70 (5.7%)	0/61	69/670 (10.3%)	27/402 (6.7%)	0/47	0/14	27/463 (5.8%)	7/31 (22.6%)	–	–	7/31 (22.6%)

County in Estonia where the small mammals were collected	Tick species attached to small mammals^a^								Overall total
	*Apodemus agrarius* (striped field mouse)				*Microtus subterranius* (pine vole)				
	*IR*	*IT*	*IP*	Total	*IR*	*IT*	*IP*	Total	
Järvamaa	–	–	–	–	–	–	–	–	25/325 (7.7%)
Lääne-Virumaa	–	–	–	–	–	–	–	–	17/209 (8.1%)
Tartumaa	–	–	–	–	0/1	–	0/1	0/2	4/152 (2.6%)
Pärnumaa	–	–	–	–	–	–	–	–	44/227 (19.4%)
Saaremaa	0/20	–	–	0/20	–	–	–	–	13/273 (4.8%)
Total	0/20	–	–	0/20	0/1	–	0/1	0/2	103/1186 (8.7%)

*IR*, *Ixodes ricinus*; *IT*, *Ixodes trianguliceps*; *IP*, *Ixodes persulcatus*; *Rh*, *Rickettsia helvetica*; *Ca*Ru, *Candidatus* Rickettsia uralica

^a^Number of *Rickettsia*-positive ticks/total number of ticks collected at that location. In parenthesis is the percentage of *Rickettsia* species and detected *Rickettsia* species in square brackets

^b^None of the *IR* from the same animal tested positive

^c^1 *Rh*-positive *IT* from animal with 1 positive and 1 negative *IR*

^d^All positive from the same animal

**Table 2:** *Rickettsia* spp. detection in ticks collected from all small mammals trapped at the collection sites

County in Estonia where the small mammals were collected	Number of ticks infected/tested										*Rickettsia* spp. genospecies^a^	
	*I. ricinus*			*I. persulcatus*			*I. trianguliceps*			Overall total	*Candidatus* Rickettsia uralica	*Rickettsia helvetica*
	Larvae	Nymphs	Total	Larvae	Nymphs	Total	Larvae	Nymphs	Total			
Järvamaa	21/288 (7.2%)	2/14 (14.3%)	23/302 (7.6%)	0/1	–	0/1	2/19 (10.5%)	0/3	2/22 (9.1%)	25/325 (7.7%)	2/25 (8%);2/325 [0.6%]	23/25 (92%); 23/325 [7.1%]
Lääne-Virumaa	15/151 (9.9%)	1/12 (8.3%)	16/163 (10.4%)	0/1	–	0/1	1/38 (2.6%)	0/7	1/45 (2.2%)	17/209 (8.1%)	–	17/17 (100%); 17/209 [8.1%]
Tartumaa	4/52 (7.7%)	0/4	4/56 (7.1%)	0/62	0/11	0/73	–	0/6	0/6	4/152 (2.6%)	–	4/4 (100%); 4/152 [2.6%]
Pärnumaa	39/186 (21.0%)	4/15 (26.7%)	43/201 (21.4%)	–	0/1	0/1	0/17	1/8 (12.5%)	1/25 (4%)	44/227 (19.4%)	1/44 (2.3%); 1/227 [0.4%]	43/44 (97.7%); 43/227[18.9%]
Saaremaa	13/247 (5.2%)	0/24	13/271 (4.8%)	–	–	–	0/2	–	0/2	13/273 (4.8%)	–	13/13 (100%); 13/273 [4.8%]
Total all counties	92/924 (10.0%)	7/69 (10.1%)	99/993 (10.0%)	0/64	0/12	0/76	3/93 (3.2%)	1/24 (4.2%)	4/117 (3.4%)	103/1186 (8.7%)	3/103 (2.9%); 3/1186 [0.3%]	100/103 (97.1%); 100/1186 [8.4%]
Total larvae (all tick species)										95/1081 (9.44%)		
Total nymphs (all tick species)										8/105 (7.62%)		

^a^Number of positives/number of all positive samples, with percentage in parenthesis; followed by number of positives/all studied samples, with percentage in square brackets

Rickettsial DNA was detected in 8.7% (103/1186) of the studied ticks, with positivity rates between tick species varying from zero for *I. persulcatus* to 3.4% (4/117) for *I.* *trianguliceps* to 10.0% (99/993) for *I.* *ricinus* (Table 2). As animal samples were not analyzed for the presence of *Rickettsia* spp., it is unknown whether ticks acquired the pathogen* via* transstadial or transovarial transmission, co-feeding or blood meal.

Rickettsial DNA was detected in ticks from all study sites, with the lowest positivity rates in Tartumaa and Saaremaa counties (2.6 and 4.8%, respectively) and the highest rate of 19.4% in Pärnumaa county.

*Rickettsia* spp. DNA was detected in ticks collected from 56 of 314 animals belonging to three species, namely *My. glareolus* (21.8%; 36/165), *A.* *flavicollis* (13.5%; 19/141) and *S.* *araneus* (33.3%; 1/3) (Additional file 1: Table S1). The number of ticks analyzed from a single animal varied from 1 to 32, while the rates of *Rickettsia*-positive ticks varied from 4.8 to 100%. The highest positivity rate of rickettsial DNA was observed in ticks from *My. glareolus* caught in Pärnumaa county (23.8%) (Table 1).

Partial *gltaA* gene sequencing results revealed the presence of two *Rickettsia* species: *R.* *helvetica* and *Ca.* R. uralica. *Rickettsia helvetica* DNA was detected in the majority of *Rickettsia*-positive tick samples (97.1%; 100/103). It was detected in 9.97% (99/993) of *I. ricinus* and in one of 117 *I. trianguliceps*. It is noteworthy that the *R. helvetica*-positive *I. trianguliceps* was attached to the same animal (*My. glareolus*) as *R. helvetica*-positive and -negative *I. ricinus* (Table 1). *Rickettsia helvetica* DNA was detected in ticks removed from yellow-necked mice, bank voles and common shrews at all study locations (Table 1).

Another *Rickettsia* species was identified as *Ca.* R. uralica*.* It was detected in three *I. trianguliceps* ticks removed from two bank voles collected in Pärnumaa and Järvamaa counties, respectively. The total positivity rate of *Ca*. R. uralica in *I. trianguliceps* was 2.9% (3/117); *Ca.* R uralica was not detected in *I. ricinus* (Tables 1, 2).

To confirm species identity and also to reveal possible nucleotide sequence variability within the detected *Rickettsia* species, we sequenced the partial *ompB* genes of 20 samples (all 3 samples with *Ca.* R. uralica and 17 samples with *R.* *helvetica*) and the partial *sca4* genes of nine samples (all 3 samples with *Ca.* R. uralica and 6 samples with *R.* *helvetica*). All sequenced *R.* *helvetica* partial gene fragments were identical to each other as well as to those previously detected in questing ticks from Estonia [12]. Sequences of *gltA*, *ompB* and *sca4* gene fragments amplified from all *Ca.* R. uralica-positive samples were 100% identical to each other; the *gltA* and *sca4* gene fragments were also 100% identical to initial sequences reported from Siberia (Genbank accession numbers KR150785 and KP747665). The *ompB* gene fragment differed in one nucleotide base, giving 99.9% similarity to the Siberian *Ca.* R*.* uralica partial *ompB* sequence (Genbank accession number KR150780) [7].

## Discussion

In this study, ticks of the generalist species *I. ricinus* and *I. persulcatus*, as well as nidicolous *I. trianguliceps*, all attached to small mammals, were analyzed for the presence of vector-borne *Rickettsia* spp., including a species not previously reported in Europe.

Many studies have focused on the circulation of *Rickettsia* species in the environment in terms of their vectors, ticks and fleas, as well as in vector-associated mammals, and the possible presence of a *Rickettsia *reservoir [18, 20, 25–27]. In the course of screening vector arthropods and their hosts, an increasing number of new “*Candidatus*” *Rickettsia* species have been identified [7, 13]. To date, however, the connection between mammals and rickettsiae has received little attention. Xenodiagnosis studies have shown negative results for *R. helvetica* [20]. In addition, the percentage of collecting *Rickettsia* spp. from the positive host is low, as described by Tomassone et al. [21]. Additional studies are required to determine the relationship between rodents and rickettsiae, the bacteremia duration, the distribution and natural cycle of *Rickettsia* spp. and the association of *Rickettsia* spp. with different arthropod vectors. Also, further research should aim to identify potential reservoir hosts and determine how* Rickettsia* spp. are maintained in nature.

To our knowledge, this study is the first to report the detection of a newly described species, *Ca.* R. uralica, in Europe. In this study, the genospecies was detected only in *I. trianguliceps* ticks removed from voles, which is in agreement with the first report of *Ca.* R. uralica from Siberia in which resemblance of* Ca.* R. uralica to* I. trianguliceps* was shown [7]. The authors of that study claim that the same *Rickettsia* variant had been previously detected in *Myodes rutilus* (northern red-backed voles) and *S. araneus*, both of which are also present in Estonia. Together with *I. trianguliceps* ticks, these small mammals might play a role in the circulation of this *Rickettsia* species in nature. Despite the genetic clustering of this newly described *Rickettsia* within the spotted-fever group, the pathogenic potential of *Ca.* R. uralica for domestic and wild mammals, pets or humans remains to be studied.

Although spotted fever rickettsioses are known to be emerging diseases that are spreading across the globe, reports of diseases due to *R.* *helvetica* infections in humans are scarce. Serological or molecular tools have been used to detect *R.* *helvetica* infection in samples collected from patients with suspected Lyme neuroborreliosis in the Netherlands [10], from those manifesting unexplained fever following a tick bite in France and Italy [11] and in those with rash, febrile illness and meningitis in Sweden [8, 9]. *Rickettsia helvetica*, a tick-borne rickettsiae species, is also frequently detected in Europe and Asia [2, 28, 29], being reported to be the prevalent *Rickettsia* species in specific regions, such as Germany [30], Slovakia [31] and Sakhalin Island in Russia [29]. Estonia is also a predominant region in terms prevalence of *Rickettsia* species, as evidenced by > 95% of all *Rickettsia* species detected in a questing study [12] and in rodent-attached ticks in the present study being *R. helvetica*. While there are no clinical reports of illness caused by *R. helvetica* in Estonia to date, the detection of this tick-borne pathogen (TBP) at positivity rates within tick population similar to the positivity rate of 23.3% for *Borrelia burgdorferi* (*s. l.*) (I. Golovljova and J. Geller, personal communications) suggests that *R. helvetica* should be considered during surveillance for tick-borne diseases in Lyme borreliosis-endemic regions.

Rickettsial DNA was detected in 8.7% of all investigated attached ticks and in 10.0% of *I. ricinus*, compared to 3.4% in *I. trianguliceps*. High rates of detection of rickettsial DNA in rodent-attached *I. ricinus* were also recently reported from Lithuania [26] where 22.6% of individually tested larvae (maximum likelihood estimation 26.5%) were positive for *Rickettsia* spp.

Several TBPs, such as *Anaplasma phagocytophilum* [32], *Neoehrlichia mikurensis* and *Babesia microti* [33], *Francisella tularensis* [34] have been detected in nidicolous rodent specialists *I. trianguliceps* ticks removed from small mammals. As reported by Igolkina et al. [7], SFG *Rickettsia* was found in 41.2% (14/34) of analyzed *I. trianguliceps* ticks feeding on voles in Western Siberia, which is significantly higher than the results reported here in our study (3.4%, 4/117). Nevertheless, the role of *I. trianguliceps* in the circulation and maintenance of TBPs is still largely unknown as is its importance and participation in the transmission of pathogens between ticks and rodent hosts.

In our study, the absence of rickettsial DNA in rodent-attached *I. persulcatus* larvae (0/64) and nymphs (0/12) could be explained by the relatively small number of *I. persulcatus* covered in the current study. However, several *Rickettsia* species, such as *Ca.* R. tarasevichiae (1/530, 0.2%) and *R. helvetica* (8/530, 1.5%) were previously reported in unfed questing *I. persulcatus* ticks in Estonia [12].

We mainly found rickettsial DNA in ticks removed from *My. glareolus* and *A. flavicollis*, although it was also presented in some ticks collected from several *S. araneus*. There are reports of the detection of *R. helvetica* in various small- to large-sized wild mammal samples from Lithuania [35], the Netherlands and Germany [18, 30, 36] and also in *Erithacus rubecula* (European robins) and *Prunella modularis* (dunnocks) from Hungary [37]; however, the significance of these animals in the transmission and maintenance cycle of *Rickettsia* is still debatable [20]. The *Rickettsia* spp. infection rates in ticks removed from the same animal varied from 4.8 to 100%, most likely indicating that the ectoparasites might acquire these pathogens not only during blood meals on these animals, but also through previous infections by transstadial, transovarial or horizontal transmission [38]. However, as there were no animal samples tested for the presence of rickettsial DNA in the current study, there is no compelling evidence on whether ticks of this study could have acquired the detected *Rickettsia* through feeding.

Surprisingly, 42.7% (44/103) of all *Rickettsia*-positive ticks were removed from rodents caught in Pärnumaa county. Although this region was not covered in the previous study on *Rickettsia* spp. in questing ticks in Estonia [12], a high rate (28%) of *Rickettsia* DNA was also detected in questing ticks in Pärnumaa (M. Vikentjeva, J. Geller, I. Golovljova, unpublished observations). Interestingly, this region has previously not shown such high infection rates with any TBP [39–41]. However, our longitudinal observations on ticks indicate that the local environment and climate of western coastal Estonia may provide favorable conditions for tick population maintenance and survival, as ticks have always been abundant in these areas (I. Golovljova, unpublished observations).

## Conclusion

The results of our study show a higher rate of positivity of *Rickettsia* spp. in ticks from small mammals compared to ones obtained previously in questing ticks. The high *Rickettsia* positivity rate in larvae might indicate a transovarial transmission of *R. helvetica* and the possibility of successful co-feeding transmission while feeding on the same host. *Rickettsia helvetica* was the most prevalent species and was most frequently detected in *I. ricinus* ticks, which are considered to be its main vector and the natural reservoir host. This study also provides the first report on the presence of the novel *Rickettsia* species *Ca*. R. uralica, initially reported from Siberian regions of Russia, in Estonian populations of *I. trianguliceps*.

## Supplementary Information

**Additional file 1: Table S1.***Rickettsia* spp. in ticks from infested small mammal species.

## Data Availability

All additional data associated with this study can be obtained from the corresponding author on reasonable request. Unique sequences of *Candidatus* Rickettsia uralica obtained during this study were submitted to GenBank database (https://www.ncbi.nlm.nih.gov/genbank/) under accession numbers MT063090–MT063092.

## Abbreviations

ITS2: Internal transcribed spacer 2
SFG: Spotted fever group
TBP: Tick-borne pathogens
