# Supplementary material for: Rickettsia spp. in rodent-attached ticks in Estonia and first evidence of spotted fever group Rickettsia species Candidatus Rickettsia uralica in Europe
Source: Parasit Vectors. 2021 Jan 20;14:65. doi: 10.1186/s13071-020-04564-7 (PMC7818765; doi:10.1186/s13071-020-04564-7)
Supplement: Supplementary file 1 — Additional file 1: Table S1.Rickettsia spp. in ticks from infested small mammal species. [file 13071_2020_4564_MOESM1_ESM.docx]

Table S1. *Rickettsia* spp. in ticks from infested small mammal species.

| Animal species | Animal collection county | № of infested animals/  № of animals with at least one *Rickettsia* positive tick (% of rodents infested by positive tick(s)) | Total № of ticks removed from infested rodents | № of *Rickettsia* positive ticks (total № ticks removed from the animals with positive tick(s)) |
| --- | --- | --- | --- | --- |
| *My. glareolus* | Järvamaa | 49/6 (12,2%) | 234 | 23 (42) |
|  | Lääne-Virumaa | 38/6 (15,8%) | 102 | 8 (35) |
|  | Tartumaa** | 28/1 (3,6%) | 100 | 1 (6) |
|  | Pärnumaa* | 37/18 (48,6%) | 122 | 29 (93) |
|  | Saaremaa | 13/5 (38,5%) | 112 | 8 (58) |
|  | ***TOTAL*** | ***165/36 (21,8%)*** | ***670*** | ***69 (234)*** |
| *A. flavicollis* | Järvamaa | 23/2 (8,7%) | 81 | 2 (3) |
|  | Lääne-Virumaa | 52/6 (11,5%) | 107 | 9 (19) |
|  | Tartumaa** | 18/3 (16,7%) | 50 | 3 (6) |
|  | Pärnumaa* | 17/6 (35,3%) | 84 | 8 (37) |
|  | Saaremaa | 31/2 (6,5%) | 141 | 5 (26) |
|  | ***TOTAL*** | ***141/19 (13,5%)*** | ***463*** | ***27 (91)*** |
| *A. agrarius* | Saaremaa | 3/0 (0,0%) | 20 | 0 (0) |
|  | ***TOTAL*** | ***3/0 (0,0%)*** | ***20*** | ***0 (0)*** |
| *S. araneus* | Järvamaa | 2/0 (0,0%) | 10 | 0 (0) |
|  | Pärnumaa* | 1/1 (100%) | 21 | 7 (21) |
|  | ***TOTAL*** | ***3/1 (33,3%)*** | ***31*** | ***7 (21)*** |
| *M. subterranius* | Tartumaa** | 2/0 (0,0%) | 2 | 0 (0) |
|  | ***TOTAL*** | ***2/0 (0,0%)*** | ***2*** | ***0 (0)*** |
| TOTAL | | **314/56 (17,8%)** | **1186** | **103 (346)** |

* - 2012 only, ** - 2013 and 2014

*A. – Apodemus*; *My. – Myodes; S. – Sorex; M. – Microtus*
